# Untying the Precise Impact of COVID-19 Policy on Social Distancing Behavior

**DOI:** 10.3390/ijerph18030896

**Published:** 2021-01-21

**Authors:** Fakhar Shahzad, Jianguo Du, Imran Khan, Zeeshan Ahmad, Muhammad Shahbaz

**Affiliations:** 1 School of Management, Jiangsu University, Zhenjiang 212013, China; 2 Department of Management Sciences, The Islamia University of Bahawalpur, Punjab 63100, Pakistan; dr.imran.khan@outlook.com; 3 Department of Business Administration, Air University, Multan 66000, Pakistan; zeeshan.ahmad@aumc.edu.pk; 4 Lyallpur Business School, Government College University Faisalabad, Punjab 38000, Pakistan; shahbaz755@yahoo.com

**Keywords:** COVID-19, social distancing, healthcare, policies

## Abstract

Social distancing has manifold effects and is used as a non-pharmacological measure to respond to pandemic situations such as the novel coronavirus (COVID-19), especially in the absence of vaccines and other useful antiviral drugs. Governments around the globe have adopted and implemented a series of social distancing strategies. The efficacy of various policies and their comparative influence on mechanisms led by public actions and adoptions have not been examined. The differences in types and effective dates of various social distancing policies in various provinces/territories of Pakistan constitute a pure ground to examine the causal effects of each COVID-19 policy. Using the location trends and population movement data released by Google, a quasi-experimental method was used to measure the impact of the government’s various social distancing policies on the people’s existence at home and their outside social mobility. Based on the magnitude and importance of policy influences, this research ranked six social distancing policies whose influence exceeded the effect of voluntary behavior. Our research outcomes describe that the trend of staying at home was firmly pushed by state-wide home order rather than necessary business closings and policies that were associated with public gathering restrictions. Strong government policies have a strong causal effect on reducing social interactions.

## 1. Introduction

The ongoing coronavirus (COVID-19) pandemic is caused by infection with acute respiratory syndrome coronavirus 2 (SARS-CoV-2). On 31 December 2019, a novel coronavirus outbreak occurred in Wuhan, Hubei, China [1] and was declared a global pandemic by the World Health Organization (WHO) on 11 March 2020. The increase in infection index has affected all WHO regions [2]. Since the Chinese authorities reported the first case of severe pneumonia to the WHO, the epidemic developed rapidly within 14 weeks [3]. The core of a contagious infection is its infectiousness—that it could be transmitted both directly and indirectly from one person to more [4]. If one person with this disease cannot transmit it to multiple people, it would gradually disappear [5]. Governments worldwide are threatened with extinction by COVID-19 and are compelled to adopt specific steps to prevent the spread of disease [6,7].

Pharmacological measures, such as vaccines and drugs, and non-pharmacological measures, such as hand washing, access to safe drinking water, and social distancing, are two core measures to control, prevent, and eliminate contagious diseases [2]. With the emergence of new infections, only non-pharmacological interventions (NPI) remain to control the infection until pharmacological options are available. Humans have an ancient but very effective way of dealing with outbreaks of infectious diseases—social distancing. Public participation is essential to reduce transmission and prevent the unprecedented intensity of a global pandemic. Governments worldwide have adopted social distancing as part of their multilevel strategies to deal with COVID-19 [8]. Social media also plays a very important role in promoting the governmental policies [9]. Social distancing refers to minimizing physical contact between individuals. From a public health perspective, the social distance will reduce or interrupt the spread of infectious diseases, especially those that spread rapidly through air droplets and contact. Social distancing applies to the individual level, including isolation of patients, isolation of contacts, and to stay-at-home advice [10].

Social distance is an incredibly useful measure aimed at reducing the propagation of viruses through air droplets. The droplets produced by coughing, sneezing, or forced speaking have a specified distance of transmission that can be reduced by keeping a distance. Wearing masks, washing hands frequently, and disinfecting with alcohol also help prevent human-to-human transmission of the virus [5,11]. Countries (such as China, Italy, Spain) implemented social isolation by imposing blockades (in specific regions or the entire country). In contrast, other countries (such as the Netherlands, Sweden, the United Kingdom, and the United States) adopted less stringent social isolation measures. So far, it is unclear how long the social distancing measures will last [12]. There is solid proof that social distance played an important role in containing the first wave of COVID-19 in China, and the most recent data suggest that this approach was effective in other countries as well [11,13,14].

However, the comparative influence of social awareness and policy intervention has yet to be revealed for the current pandemic. Besides, some additional policies have been implemented to enhance social distance, some of which may have unintended effects. Consequently, determining effective strategies can assist policy makers in effectively reacting to the pandemic. A study [15] used epidemiological models to explore whether similar mitigation and suppression strategies are equally reasonable in low- and middle-income countries. They concluded that in high-income countries such as the United States and Mainland China, social distancing procedures are expected to save a substantial number of lives, while in low-income and low-middle-income countries, the estimated benefits of social distancing are much lower; Pakistan is one of them. Therefore, this study referred to [15] that the dynamic impact of mobility on individual cases is roughly similar across counties and cities, but due to differences in policy implementation and civic behavior, mortality is different.

So far, relatively few studies have estimated the direct influence of movement rates on the infection rate of COVID-19. Previous research tended to focus on NPI policies—especially the impact of shelter-in-place orders (SIPO) on the transmission of COVID-19—instead of whether the direct impact of the channel of various NPIs on the growth of COVID-19 cases has a substantial beneficial impact [16,17,18]. The prior studies did not investigate the role of fluidity but assumed that the impact of NPIs on COVID-19 incidents was arbitrated through the channel of NPIs, decreasing fluidity and thereby reducing infection. This study used Pakistan’s daily province/territory-level changes in six different intervention policies (declaring a health emergency, education institutions closure, transportation ban, lockdown, shut down cinema halls, bans of public gatherings) to inspect their underlying influence on various social indicators on keeping a distance. Moreover, a lockdown lifting policy was also incorporated to evaluate the counter effect.

## 2. Material and Method

### 2.1. Mobility Trend Data

We collected and used daily human mobility indicators published by Google (Google Inc, Menlo Park, CA, USA) in several places, such as residential areas, grocery stores and pharmacies, parks, retail and entertainment venues, transit stations, and workplaces. Google collects daily anonymous location data of users’ movement trends in different geographic locations and at different times from those who intentionally enable location settings on their devices. Google uses the same data on Google Maps to track human traffic in different locations. The data were released in the form of a comma-separated values (CSV) file on 30 May 2020, including the global mobility data on the already mentioned six different places from 15 February to 25 May. These global data also included specific data for each province/territory of Pakistan. COVID-19 prevention policies are mostly implemented at the provincial/territorial level; therefore, our analysis was based on the provincial/territorial level. We aggregated the data points because each trend contained multiple data points every day to get a unique movement index every day. Google explained in the data file that these data only include those who bring their phones/gadgets to places where location services are turned on. Most of the provincial/territorial data of movement was available for the reported time (101 days), providing us with 606 observations. However, we could not resume the three observations for Azad Jammu and Kashmir (AJK). Due to the missing values in dataset, we gave up two territories of Pakistan in our final analysis, including the Federally Administered Tribal Area and Gilgit-Baltistan.

The objectives of COVID-19 prevention policies can be measured in multiple ways. The main focus was to assess the impact of the COVID-19 prevention policy in reducing personal mobility and restricting people to their homes. Current research uses the time people spend in their place of residence (present as home/stay-at-home variable) as a proxy of the success of the policy. In addition, to encouraging people to stay home, banning traffic and closing educational institutions also keeps people away from crowded places, even if they have already left home. The purpose of this research was to evaluate the impact of the COVID-19 prevention policies on population changes in five locations (such as parks, grocery stores and pharmacies, workplaces, retail and recreation areas, and transit stations). Google data explains that these places include all gathering places, such as places of worship; therefore, we focused on analyzing the impact of the prevention policies on staying at home.

### 2.2. Province/Territory Policy Data

For the collection of policy-related data, this study used the original documents issued by the provincial/regional government, which are reported by national agencies and different news channels (such as Samma TV, Dawn News, Geo TV, ARY News, PTV news, etc.). The issuance/implementation date mentioned in the policy letters were considered the starting day of policy implementation.

Consistent with the research goals, only those policies that forced people to avoid social gatherings were included in the study. Therefore, this research reports six different policy letters issued by provincial/territorial governments, including declarations of health emergencies, closure of educational institutions, prohibition of transportation, lockdown, closure of cinemas, and prohibition of public gatherings. In addition, a lockdown lifting policy was also incorporated to evaluate the counter effect.

**Figure 1:**
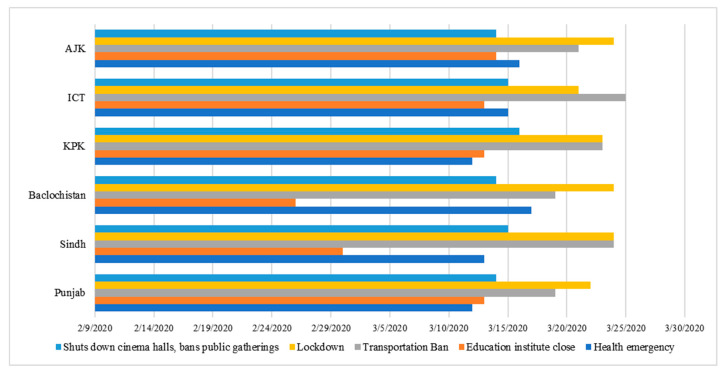
Social distancing policies (adoption) during COVID-19 by provinces/territories of Pakistan. [AJK = Azad Jammu and Kashmir; ICT = Islamabad Capital Territory; KPK = Khyber Pakhtunkhwa (x-axis = Province/state; y-axis = Starting date of policy implementation)].

Similarly, regional governments have also adopted other policies to prevent COVID-19, such as the Ehsaas Relief Program, COVID-19 emergency relief in Sindh, etc., in which governments provided monthly allowances to poor families, allowing them to stay at home or receive treatment, or compulsory isolation of travelers, which we did not cover in the current study. The summary of the policy adoption time of each province/region is available in Figure 1. These policies were grouped because of the short differences in the execution/release time of these policies. Health emergencies, closures of educational institutions and cinemas, bans on public gatherings, traffic bans and lockdowns were divided into two variables. In most provinces/regions, their implementation dates differed by only a few days.

### 2.3. Experimental Design

This study used a quasi-experimental method (difference-in-difference), which is a commonly used social science method to measure policy effects [11,19]. Specifically, this study compared the change in daily visits from different locations in different provinces/territories that adopted COVID-19 prevention policies, particularly before and after implementing these policies.

(1) $$Y_{pt}=\alpha +X_{pt}\beta +\lambda_{p}+\varepsilon_{pt}$$

where *Y* is the changes in visits at various places including (province/state wise) present at home, parks, grocery and pharmacy, transit stations, retail and recreation, and workplaces. *X* is the matrix of COVID-19 prevention policies, introduced in data section. *λ* is the sets of a day of the month fixed effect. We defined a dummy variable for each policy. If a given province/region implemented the policy, it was set to 1, otherwise it was set to 0. Due to the serial correlation problem in the visit changes within the same province/territory, we used standard error clustering at the provincial/territorial level to overcome this problem. For provinces/territory without these policies, these variables were set to 0.

## 3. Results

Table 1 shows the outcomes for the impact of COVID-19 prevention policies presented above on the daily visits at several places in different provinces/territories of Pakistan. This study’s primary concern was to focus on how COVID-19 prevention policies restricted people to stay at home, shown in column two of Table 1. Results report that province/territory-wise lockdown and a ban on transportation orders significantly restricted people to present at home. This policy’s highest impact was reported in Islamabad Capital Territory (ICT), followed by Sindh and Punjab. The possible reason for the high impact in ICT may be the education level. ICT is the capital of Pakistan and has a literacy rate of more than 85%, which is high compared to other provinces/territories of Pakistan (Punjab and Sindh, about 60%).

Closure of educational institutions policy and the announcement of health emergency had a significantly positive effect on restricting people at home. However, this policy’s effect size was smaller than the lockdown and transportation ban policy (about 6 times lower). The closure of educational institutions and the announcement of health emergencies were insignificant in Balochistan because Balochistan’s literacy rate is the lowest in the county.

**Figure A1:**
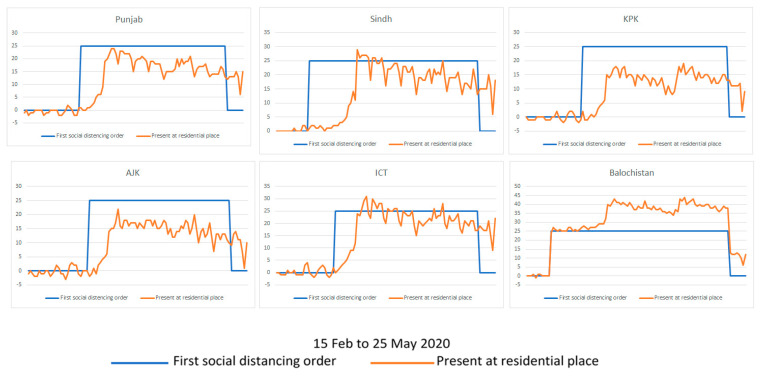
Tendencies in presence at a residential place and the start date of the first social distancing order in each province/territory.

**Figure A2:**
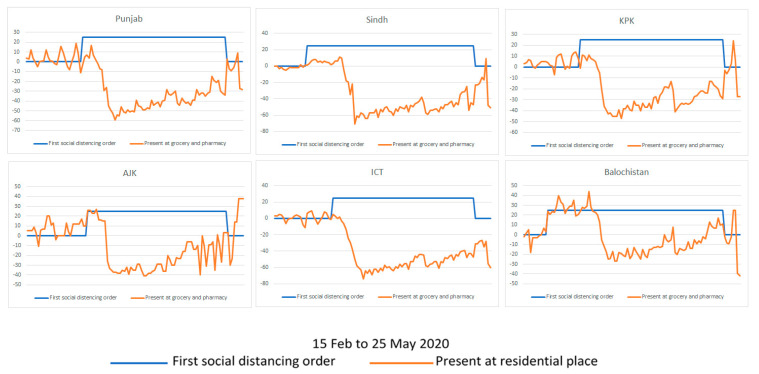
Tendencies in presence at a grocery or pharmacy and the start date of the first social distancing order in each province/territory.

**Figure A3:**
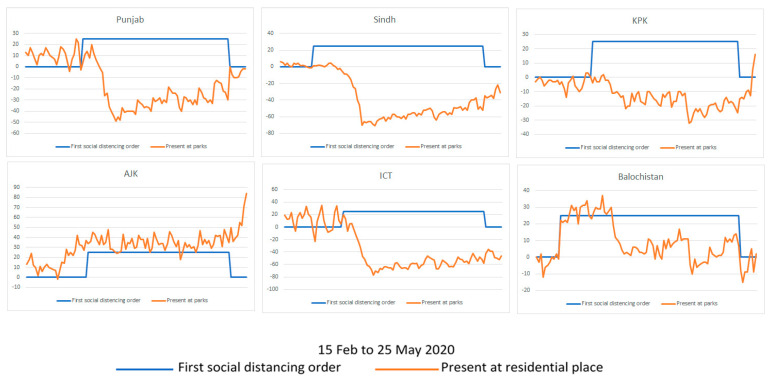
Tendencies in presence at parks and the start date of the first social distancing order in each province/territory.

**Figure A4:**
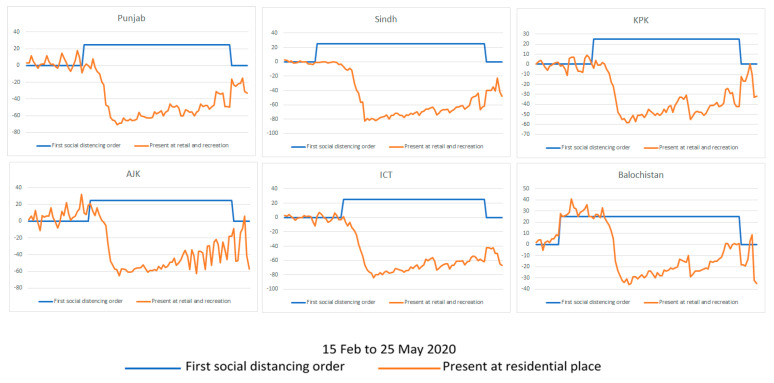
Tendencies in presence at retail and recreation and the start date of the first social distancing order in each province/territory.

**Figure A5:**
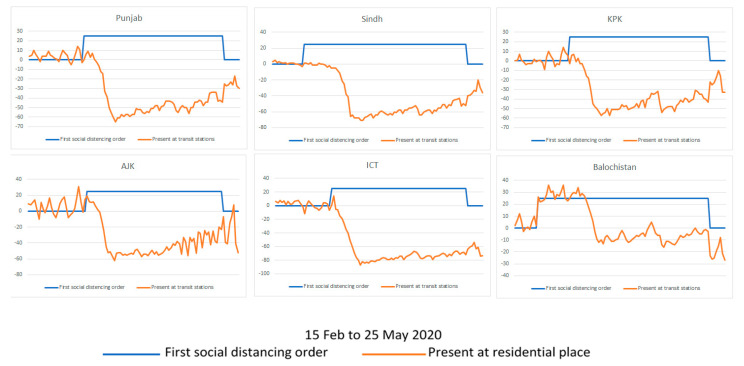
Tendencies in presence at transit stations and the start date of the first social distancing order in each province/territory.

**Figure A6:**
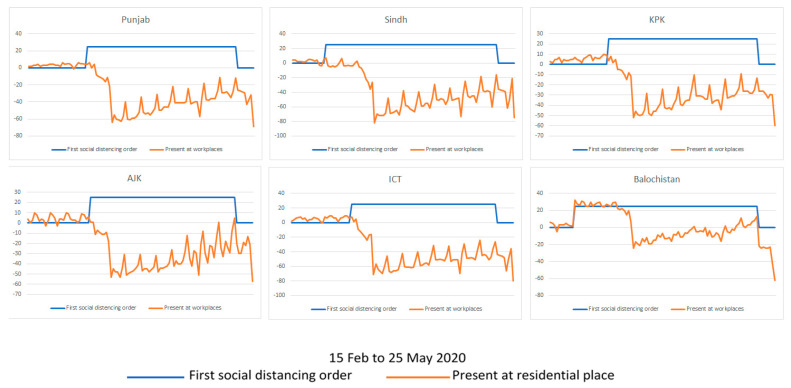
Tendencies in presence at the workplace and the start date of the first social distancing order in each province/territory.

The rest of the columns in Table 1 show the results of a change in visits from places other than home, among them the retail and recreation settings, parks, groceries and pharmacies, transit stations, and workplace settings. Interestingly, the results show a reduction of people present at all out of home places, which provides evidence that the policies’ findings to restrict people to stay at home are not spurious. We used a regression model to measure results. R-squared is a statistical measurement that represents the proportion of variance of a dependent variable. This dependent variable can be explained by one independent variable or multiple variables in the regression model. The value of R-squared indicates the fitness of the model. Results presented in Appendix A (Figure A1, Figure A2, Figure A3, Figure A4, Figure A5 and Figure A6) support this hypothesis.

These data show the impact of different social distancing policies on population movement in different provinces/territories of Pakistan. After the implementation of the first social distancing policy, the mobility of the population changed significantly. Figure A1 shows the increase in the number of people at home after the implementation of the first social distancing policy. Figure A2 shows the change in the number of people in grocery stores and pharmacies after the implementation of the social distancing policy. Figure A3 shows the park traffic after the implementation of the first social distancing policy. Figure A4 shows the changes in the number of people in retail and entertainment venues. Figure A5 shows the tendencies in people’s presence at transit stations. Finally, Figure A6 shows a tendency in the number of people in the workplace after the implementation of the first social distancing policy.

## 4. Discussion

It has been concluded from the results that transportation ban and lockdown policy most effectively restricted people’s visits to retail and recreation places in all provinces/territories of Pakistan except ICT, where people’s presence increased at these places. Moreover, announcements of health emergencies and closure of educational institutions policies also reduced people’s presence at retail and recreation places in all provinces/territories of Pakistan except ICT, where people’s presence increased at these places. The possible reason for the increased number of people present at retail and recreation places in ICT may have been the upcoming uncertainty of the situation, requiring people to store eatables. Therefore, the residents of ICT frequently visited retail places to purchase a necessary item for storage.

The effect of health emergency declarations and educational institutions’ closure policies significantly reduced the presence of people at parks in Sindh and ICT. These policies were ineffective in other provinces/territories in restricting people’s visits to parks, except in AJK, where people increased their visits to parks. The effect of transportation bans and lockdowns significantly reduced the number of people visiting parks in all provinces/territories of Pakistan except ICT. The possible reason for such behavior by the people of AJK may have been the low volume of COVID-19 cases in AJK. The highest impact of a transportation ban and lockdown policy on restricting people’s visits to parks was in ICT, followed by Sindh and Punjab.

The effect of educational institutions’ closure policy and health emergency announcements was insignificant in restricting people’s visits to groceries and pharmacies in all provinces/territories of Pakistan because these policies did not impact grocery and pharmacy work. The effect of a transportation ban policy and lockdown on restricting people’s visits to a grocery or pharmacy was significant in Pakistan’s territories/provinces. The highest impact of a transportation ban and lockdown policy on restricting people’s visits to a pharmacy or grocery was in ICT, followed by Sindh and Punjab.

The effect of educational institutions’ closure policy and health emergency declarations was insignificant in restricting people’s visits to transit stations in 50% of Pakistan’s provinces/territories. The highest impact of educational institutions’ closure policy and health emergency on restricting people’s visits to transit stations was in ICT, followed by Sindh and KPK. Moreover, a transportation ban policy and lockdown on restricting people’s visits to transit stations were significant in Pakistan’s territories/provinces. The highest impact of transportation ban policy and lockdown on restricting people’s visits to transit stations was ICT, followed by Sindh and Punjab.

The effect of educational institutions’ closure policy and health emergency announcements on restricting people’s visits to workplaces was significant in all territories/provinces of Pakistan except Baluchistan. Moreover, a transportation ban policy and lockdown on restricting people’s visits to workplaces were significant in Pakistan’s territories/provinces. The highest impact of transportation ban policy and lockdown on restricting people’s visits to workplaces was in KPK, followed by ICT and Sindh.

Among all the significant coefficients present in Table 1, the most-pronounced ones were related to lockdown and a transportation ban, which indicates that these policies were the most effective policies for restricting people to stay at home and enhancing social distancing. These results are also in accordance with the study of [11]. Although other policies, including announcing health emergencies and closing educational institutions, significantly influenced people’s restriction to stay at home in most provinces/territories, the effect size was small compared to lockdown and transportation ban policies.

Surprisingly, the closure of educational institutions and health emergency, transpiration ban, and lockdown policies in ICT was positive and significant at decreasing people’s presence at retail and recreation places—the possible reason for this positive effect may be uncertainty, which influenced people to store eatables for lockdown. Therefore, the effect of these policies on restricting people at home was highest in ICT. Moreover, other policy measures such as the closure of educational institutions and the declaration of a health emergency also restricted people’s visits to places such as retail and recreation areas, parks, groceries and pharmacies, transit stations, and workplaces, but its causal effect on keeping people at home seems less critical.

This study also evaluates Eid’s effect (a primary event of Muslims; celebrated on 24 May 2020) on people at different places. Before the celebrations of Eid, the supreme court of Pakistan lifted the lockdown and directed the government to open markets and super malls on 18 May 2020. This lift in lockdown policy significantly affected people’s travel to different locations. The people’s presence at home reduced significantly in all provinces/territories in Pakistan. Moreover, the lift in lockdown policy encouraged people to visit different places other than home. This relaxation in the public gathering bans heavily increased the number of COVID-19 cases in Pakistan. Total COVID-19 confirmed cases jumped more than double from 43,966 to 93,983, and total deaths also jumped more than double from 939 to 1935 in just 19 days (18 May to 6 June). This toll confirms that the policies restricting people to stay at home significantly protected people from COVID-19.

## 5. Implications

Regarding this study’s implications, the present study results enrich the current knowledge of COVID-19 and social distancing and provide a bird’s-eye view for policymakers to develop and implement pandemic control policy decisions. Moreover, we concluded that people must be careful if this early pandemic’s effects are generalized in later stages and future potential waves of the pandemic. Explicitly, this study posited that the findings might not indicate that more simplistic policies such as announcements of health emergencies and educational institutions’ closure inefficiently trigger stay-at-home effects. However, much of such interventions’ social distancing potential is already consumed in non-policy-driven improvements—likely triggered by social awareness. The pandemic will likely last longer, and the concept of not going out of the home will start diminishing, making policies such as health emergency and closure of educational institutions more useful in later waves of COVID-19 [20].

## 6. Future Directions

Regardless of the important implications, this study has a few limitations. First, this study used initial documents issued by the provincial/territorial governments and reported by different news channels; the only other source that could also be incorporated was Google’s community mobility reports, which use their accounts’ location history. Second, we only used six different policy letters but did not include their effects on Pakistan’s religious activities as it is an Islamic country, and men visit Masjid (mosque) five times a day. The policy effects on Masjid visits and religious gatherings’ role were not considered; future research can consider several religious activities’ policies. Third, future research should also incorporate the effectiveness of “smart lockdown”, a term introduced by Pakistan’s government to restrict a specific locality of people instead of the whole territory to stay at home. Fourth, this study did not correlate data on people’s movement with epidemiological records because we have only used the data from people who regularly carry a mobile device with active GPS tracking and Google services enabled. Therefore, researchers can expand this study by examining epidemiological records. Finally, as it is likely that the pandemic will last longer, if there are multiple waves of COVID-19 around the globe, it would be interesting to see whether these policies will bring either further economic and human loss or will tackle the pandemic more smartly and without the lockdown of economic hubs.

## 7. Conclusions

Our findings prove the efficacy of various province/territory policies to limit out-of-home social contact during the early stages of the epidemic of COVID-19. When the pandemic intensified sharply in early March 2020 and the province/territory started enforcing residential directives, such as lockdown policy, ban on transportation, closure of educational institutions/health emergency, the beneficial impacts of “stay home, stay safe” directives correlated with these orders. Our findings indicate that increases in stay-at-home policy and fewer social interactions were motivated by a mixture of regulation and voluntary interventions and point to the apparent causal effect of province/territory complete lockdown and the more modest impact of the ban of transportation and the closure of educational institutions. 

## Figures and Tables

**Table 1:** Effect of COVID-19 prevention policies on community mobility.

Factors	Stay at Home	Present at Retail and Recreation	Present at Parks	Present at Grocery and Pharmacy	Present at Transit Station	Present at Workplace
**Azad Jammu and Kashmir (AJK)**						
EDU_health	3.39 **	−8.85	21.49 **	9.03	−8.01	−17.13 **
Transp_Lockdown	15.34 **	−55.20 **	16.66 **	−32.05 **	−51.54 **	−39.19 **
Lift_Lockdown	9.79 **	−34.63 **	36.41 **	4.43	−30.23 **	−30.93 **
R-squared	0.86	0.77 *	0.57	0.83	0.78	0.68
**Islamabad Capital Territory (ICT)**						
EDU_health	3.73 *	3.17 **	−12.28 **	−5.81	−13.26 **	−13.89 **
Transp_Lockdown	22.08 **	1.84 **	−70.05 **	−55.03 **	−75.84 **	−56.09 **
Lift_Lockdown	17.10 **	3.17 **	−55.53 **	−38.06 **	−65.39 **	−57.39 **
R-squared	0.89	0.94	0.90	0.90	0.95	0.84
**Khyber Pakhtunkhwa (KPK)**						
EDU_health	2.09 *	−7.95 **	0.08	−1.80	−7.06 **	−9.34 **
Transp_Lockdown	14.27 **	−44.90 **	−14.27 **	−35.56 **	−46.37 **	−39.54 **
Lift_Lockdown	10.27 **	−16.29 **	−3.34	−8.74 *	−23.53 **	−37.99 **
R-squared	0.89	0.86	0.60	0.88	0.90	0.81
**Balochistan**						
EDU_health	1.00	−0.27	4.73	3.91	−0.73	−0.86
Transp_Lockdown	13.08 **	−47.20 **	−16.75 **	−33.20 **	−33.64 **	−32.99 **
Lift_Lockdown	11.07 **	−18.92 **	−2.92	−5.32	−24.23 **	−33.01 **
R-squared	0.84	0.82	0.68	0.66	0.83	0.76
**Sindh**						
EDU_health	4.32 **	−15.57 **	−12.54 **	−1.68	−11.13 **	−12.92 **
Transp_Lockdown	20.21 **	−68.62 **	−58.28 **	−49.28 **	−59.88 **	−54.22 **
Lift_Lockdown	14.67 **	−38.03 **	−34.57 **	−21.97 **	−34.63 **	−47.15 **
R-squared	0.80	0.83	0.84	0.89	0.74	0.72
**Punjab**						
EDU_health	3.04 **	−8.95 **	−3.32	−0.74 *	−4.92	−8.96 *
Transp_Lockdown	18.31 **	−58.47 **	−41.65 **	−42.55 **	−53.36 **	−45.60 **
Lift_Lockdown	13.04 **	−26.60 **	−15.92 **	−11.16 **	−29.24 **	−39.84 **
R-squared	0.90	0.91	0.87	0.91	0.93	0.78

Significant at: * *p* < 0.05, ** *p* < 0.01. EDU_health = education–health; Transp_Lockdown = Transportation lockdown.

## Data Availability

This is available at: https://www.google.com/covid19/mobility/index.html?hl=en (accessed on: 06-06-2020).
